# Reliability and Validity of the KFORCE Sens^®^ Inertial Sensor for Measuring Cervical Spine Proprioception in Patients with Non-Specific Chronic Neck Pain

**DOI:** 10.3390/brainsci14121165

**Published:** 2024-11-22

**Authors:** George A. Koumantakis, Stamatina Gkouma, Christina Floka, Petros I. Tatsios, Maria Moutzouri, Vasiliki Sakellari

**Affiliations:** 1 Physiotherapy Department, School of Health & Care Sciences, University of West Attica (UNIWA), 12243 Athens, Greece; mscphys23005@uniwa.gr (S.G.); mscphys23016@uniwa.gr (C.F.); ptatsios@uniwa.gr (P.I.T.); moutzouri@uniwa.gr (M.M.); vsakellari@uniwa.gr (V.S.); 2 Laboratory of Advanced Physiotherapy (LAdPhys), Physiotherapy Department, School of Health & Care Sciences, University of West Attica (UNIWA), 12243 Athens, Greece

**Keywords:** cervical spine, chronicity, motor control, position sense, repositioning error, reproducibility

## Abstract

Background/Objectives: Patients with non-specific chronic neck pain (NSCNP) exhibit sensorimotor disturbances, with proprioception impairment considered an important aspect. The aim of this study was to assess the reliability and validity of a novel inertial sensor-based electrogoniometer (KFORCE Sens^®^) for cervical spine (CS) proprioception measurement in patients with NSCNP. Methods: The within-day intra-rater reliability of CS proprioception and its association with patient demographics and clinical status were examined in fifty-nine patients with NSCNP, aged between 25–65 years, recruited from primary care. CS proprioception was examined via angle reproduction, in angles set mid-range in the available CS range of motion (ROM) in each motion direction. The clinical status evaluation comprised the maximum and average pain intensity in the last week, disability, fear of movement/re-injury, catastrophizing, neck awareness, and CS-ROM. Reliability was assessed using the intraclass correlation coefficient (ICC_2,1_), standard error of the measurement (SEM), and minimum detectable change (MDC_95%_). Pearson’s R assessed between-measures associations. Results: CS proprioception reliability was good (ICC_2,1_ = 0.75–0.89), with low measurement error (SEM = 1.38–3.02° and MDC_95%_ = 3.83–8.38°). Correlations between participants’ CS proprioception and their clinical status or demographics were not significant. Conclusions: The reliability of CS proprioception assessment with the KFORCE Sens^®^ was good in a sample of mildly to moderately disabled patients with CNP and thus deemed suitable for further research in this field.

## 1. Introduction

Neck pain (NP) is the fourth most common cause of disability worldwide [1]. The average estimates for its prevalence are 7.6% for current prevalence, 37% for annual prevalence, and 48.5% for lifetime prevalence [1,2]. There are many NP classification systems available, with a main classification scheme based on the duration of symptoms (acute pain: ≤6 weeks, subacute: between 6 weeks and 3 months, and chronic: ≥3 months) [3].

In the absence of a lesion in the nervous system or other serious pathology, nociceptive-type mechanical pain arises from the spine or its supporting structures, muscles, and ligaments due to the presence of arthritis, degenerative lesions in the intervertebral discs, or myofascial pain [4], and it is also linked to sustained poor posture and repetitive or awkward movements [5,6]. However, longer-term disturbance of the sensorimotor system function may result in neuroplastic changes within the central nervous system (CNS), leading to chronic nociplastic-type pain [4] due to central pain-processing alterations [7,8,9]. Proprioception sense encompasses various afferent signals from peripheral tissues, collectively contributing to the sensation of body parts’ movement and orientation in space [10,11], as well as the sense of force or effort [11]. Proprioception is considered a key element in the seamless execution of pain-free movement [8]. In chronic pain conditions, head–neck proprioception acuity is considered to be influenced by altered sensorimotor control at multiple levels of pain transmission and centrally planned movement execution [8,9]. Proprioception signals, contributing to the integration of sensory information, can be affected by pain-related neuroplastic changes [12,13]. Therefore, the abnormal proprioceptive signal processing from the CNS related to motor control impairments could possibly contribute to the chronicity of pain conditions [14].

There is an array of tests to assess sensorimotor dysfunction in chronic pain states [15], with proprioception forming part of this test battery [8]. Proprioception testing encompasses various protocols; some studies focus on repositioning errors in the neutral position, while others examine errors at angles within the available range. However, the number of tests, repetitions per test, and equipment used vary between studies [16,17]. Some studies have observed significant proprioception dysfunction in patients with non-specific chronic neck pain (NSCNP) compared to healthy subjects [18,19,20,21]. Other studies have found this proprioception dysfunction to be minor [13,22,23], limited to certain proprioception measures [24] or certain movement directions [25], or pertaining to patients with CNP with neuropathic characteristics [26]. This could be because of differences in testing methods between studies and fluctuations in patient clinical status. Furthermore, clinically, significant associations of moderate levels and not in all movement directions have been demonstrated between proprioception impairment and patients’ kinesiophobia [27,28,29] and catastrophizing [29,30], while other studies have not shown such associations to be present, both in studies with cross-sectional [31] or prospective [32] designs.

For pain intensity or pain duration, most studies were not able to identify significant associations with proprioception acuity [27,28,29,32,33], with one study identifying a moderate association (r = 0.51) between pain intensity and one out of the eighteen proprioception measures reported [34]. However, in a study performed on patients with cervical spondylosis and CNP, moderate to good associations (r = 0.59–0.78) were identified between pain intensity and proprioception deficits for all four movement directions tested [35]. Only two studies have so far shown a link between proprioception dysfunction and disability. The first study reported a fair correlation (r = 0.32) between “repositioning errors” and disability, but it did not specify the significance level of this association or if it was present for all of the four movement directions tested [33]. The second study confirmed a significant moderate association (r = 0.56) for only one of the four tests used, and only for one of the two movement directions tested [29].

Improvement in tests employed and technological advancements in monitoring devices may provide a more detailed picture of the extent of proprioception dysfunction present in CNP [24,36,37,38,39]. Additionally, the relationships of proprioception dysfunction with patient demographics and condition severity characteristics require more detailed documentation, as these may vary between certain patient subgroups [13]. Therefore, the aim of this study was to assess the accuracy of a lightweight and easy-to-apply novel inertial sensor-based electrogoniometer in measuring cervical spine (CS) proprioception. It also aimed to examine its construct validity via possible associations between CS proprioception and an array of clinical status characteristics and patient demographics.

## 2. Materials and Methods

### 2.1. Sample

This study included 59 adult participants between 25 and 65 years old, with a diagnosis of NSCNP lasting for more than 3 months, referred for physical therapy in a large private physiotherapy practice. All participants were fluent in speaking and reading Greek. People who had received physical therapy in the past 6 months for their neck pain; those with neurological or psychological disorders; and those with vestibular, hearing, or uncorrected visual impairments, cervical radiculopathy, previous spinal surgery, a diagnosis of serious spinal pathology (cancer, inflammatory arthropathy, or spinal fracture), or pregnancy were excluded.

### 2.2. Ethics

The Ethics Committee of the University of West Attica, Athens, Greece, approved the protocol of this research study (approval no: 63750/4-7-2023), according to the Declaration of Helsinki. A detailed information sheet describing the aims and purposes of this study was provided to all possible participants, and those who agreed to take part filled in and signed a relevant consent form prior to their inclusion in the study.

### 2.3. Study Design

This study was of a cross-sectional design.

### 2.4. Procedures

Participants were first asked to complete their demographic and neck pain pathology characteristics (duration of current episode and history of recurrences) and the following five clinical status questionnaires. Cervical spine range of motion (CS-ROM) and CS proprioception measurements followed.

#### 2.4.1. Pain Intensity Visual Analog Scale (PI-VAS)

The PI-VAS scale assesses the intensity of pain in various pain conditions. The PI-VAS scale typically takes the form of a 10-cm-long straight horizontal line, where the left end represents no pain at all, all values to the right represent increasing pain intensity, and the extreme value at the right end of the line represents the worst possible pain [40]. The scale has been utilized among Greek patients with CNP [41]. We chose to use a PI-VAS scale with two instructions: “Average pain over the last week” and “The worst pain over the last week” [40].

#### 2.4.2. Neck Disability Index—Greek Version (NDI-GR)

The NDI is a 10-item scale designed to assess disability related to daily life activities due to neck pain (9 items), as well as the intensity of pain (1 item), by selecting one of the six available responses (Likert scale, 0–5) in each of the ten examined areas [42]. The rating ranges from zero (least restriction and no pain) to five (maximum restriction and worst pain). The overall score ranges from zero (no disability) to fifty (maximum disability). The questionnaire has been validated in Greek patients with CNP, with high reliability, validity, and internal consistency reported [43].

#### 2.4.3. Tampa Scale of Kinesiophobia—Greek Version (TSK-GR)

The TSK was created to assess fear of movement/re-injury, a frequent condition in patients with chronic pain characterized by an illogical, unusual, excessive, and debilitating fear of physical movement and activity due to a feeling of fear from a previous or potential impending injury [44]. The scale has been shown to moderately correlate with proprioception acuity in patients with NSCNP [27,28,29]. The scale consists of 17 self-report items, with each item scored on a 4-point Likert scale ranging from 1 (completely disagree) to 4 (completely agree), and the scale score ranges from 17 (no fear) to 68 (serious/pathologic level fear). The scale has been cross-culturally adapted and validated in Greek patients with chronic pain [45].

#### 2.4.4. Pain Catastrophizing Scale—Greek Version (PCS-GR)

This questionnaire quantifies the extent of negative catastrophic thoughts that an individual may be feeling as part of their pain experience. It consists of thirteen questions with five possible answers, from zero (not at all) to four (constantly). The overall score ranges from zero (no perception of catastrophizing) to fifty-two (maximum perception of catastrophizing) [46]. The questionnaire has been cross-culturally adapted and validated for the Greek population [47].

#### 2.4.5. Fremantle Neck Awareness Questionnaire—Greek Version (FreNAQ-GR)

The FreNAQ is a questionnaire recently developed to assess body awareness found to be altered in patients with NSCNP [48,49]. It consists of nine items assessing deviations in neck shape, size, position, and motor control, with each item scored on a 5-point Likert scale (0 representing ‘never’ to 4 representing ‘always’ feeling like that) and scores ranging from 0–36, with higher scores denoting greater impairment in neck body awareness. The FreNAQ has been recently cross-culturally adapted and validated in Greek patients with NSCNP [50].

#### 2.4.6. Cervical Spine Range of Motion (CS-ROM) and Proprioception Measurements

The study’s coordinator (G.A.K.) and the owner of the physiotherapy practice (P.T.) trained two members of the research team (S.G. and C.F.) in the conduct of all CS-ROM and proprioception measurements, as the former were more experienced in treating patients with neck pain and proprioception measurement methods.

All CS-ROM and proprioception measurements were conducted with the KFORCE Sens^®^ (KINVENT, Montpellier, France), a small (15 × 56 × 35 mm), lightweight (40 g) inertial sensor-based electrogoniometer with a manufacturer-reported accuracy of 3° [51]. With the aid of a customized mobile phone application, the device can transmit and display recorded data in real-time to a mobile phone via Bluetooth (2.4 GHz band) at a distance of up to 10 m. The application can then store the ROM, time, and speed data related to the movement direction measured for later display and analysis. The device has been previously used to assess wrist ROM and proprioception [52] and has not yet been used for the corresponding CS measurements.

**Figure 1:**
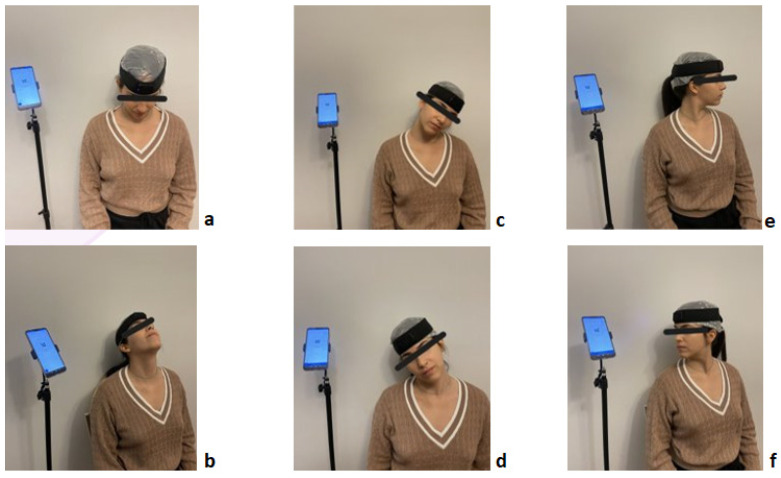
Measurements of cervical spine ROM in flexion (**a**), extension (**b**), right and left side flexion (**c**,**d**), and right and left rotation (**e**,**f**).

The measurement process initially involved measuring the full available CS-ROM in the sagittal plane (flexion and extension), the frontal plane (right and left lateral flexion), and the transverse plane (right and left rotation), assessed in random order. The measurements were taken from a seated position in a stable chair with the patient’s feet in contact with the ground, forming a right angle with the hip, and the shoulder blades supported by the chair so that the movement was exclusively performed by the cervical spine (Figure 1). Three sequential measurements of the full CS-ROM were executed per movement direction, and the highest value of the three was selected for each of the movement directions.

**Figure 2:**
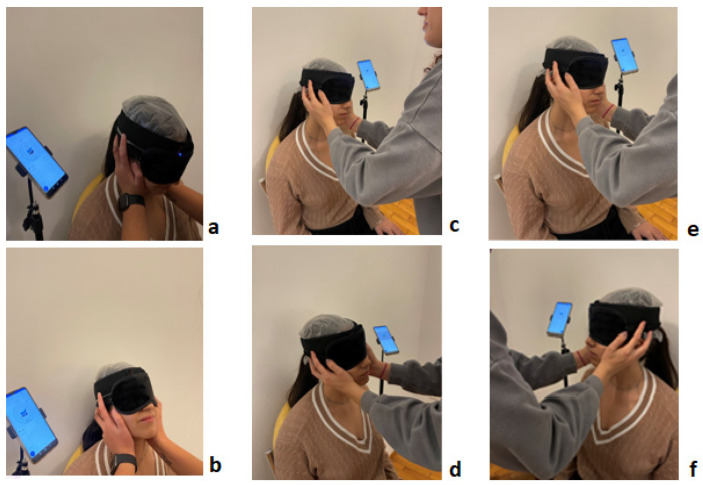
Passive cervical spine placement in the ‘target angles’ (50% of cervical spine ROM) of flexion (**a**), extension (**b**), right and left side flexion (**c**,**d**), and right and left rotation (**e**,**f**), with participant in a seated position wearing an eye mask.

For CS proprioception measurements, the participants’ ability to reproduce a pre-specified ‘target angle’ set at 50% of the full CS-ROM for each of the above six movement directions was assessed, with participants blindfolded throughout the procedure. The process followed was divided into two steps per movement direction. First, participants were passively taken from the neutral position 0° and placed at each pre-specified ‘target angle’, at which they were kept for 5 s, allowing for this position to be memorized (Figure 2), and then they were passively returned to the neutral position. As a second step, we asked participants to actively identify the pre-specified ‘target angle’ at each movement direction by performing three sequential repositioning trials at their own pace, starting from neutral, remaining in place for 2 s when they believed they had reached the ‘target angle’, and then returning to neutral. We performed CS proprioception in each movement direction immediately after the corresponding CS-ROM test, ensuring that the procedure followed the same random order to avoid possible interaction effects between measurements [53].

For the measurement of proprioceptive acuity, the absolute error (AE) index was used, calculated as the average of the absolute deviation values of the three sequential repositioning trials from the ‘target angle’ [54]. The AE index has been used as the primary outcome measure for assessing spinal proprioception and thus provides a direct comparison with other studies.

All measurements were collected in a quiet room with a stable temperature of 23 °C. The participants’ vision was occluded throughout the experiment, with their eyes covered (application of a non-photo-permeable eye mask) and the goniometer, which was mounted on a headband, firmly secured at the center of their foreheads. In all stages, the necessary hygiene measures were observed. Before the measurements began, the space was adequately ventilated, the objects (chair, KFORCE Sens^®^ sensor) were sterilized, and during the placement of the eye mask (blindfold) and the sensor stabilization strap, a disposable protective non-woven fabric was first applied to the forehead and eye areas.

### 2.5. Statistical Analysis

All analyses were performed with IBM SPSS Statistics v.29.02.00 software. The distribution of continuous variables was analyzed with the Kolmogorov–Smirnov test. All descriptive statistics of participants’ demographic details, their CS-ROM and proprioception, as well as their questionnaire scores (PI-VAS, NDI-GR, TSK-GR, PCS-GR, and FreNAQ-GR) were analytically presented, depending on the distribution of each variable. The possible influence of the anthropometric characteristics or the level of self-reported physical activity on the patient’s clinical status data (questionnaires, CS-ROM, and CS proprioception) was examined with the use of Pearson’s correlation coefficients, and that of gender was assessed with the independent samples t-test.

The within-day test–retest intra-rater reliability of the three sequentially executed ‘target angle’ repositioning trials was calculated using the two-way random effects absolute agreement single measurement intraclass correlation coefficient (ICC_2,1_) [55], the standard error of the measurement (SEM), and the minimum detectable change (MDC_95%_) [53,56]. ICCs less than 0.5 were interpreted as poor, those between 0.5 and 0.75 as moderate, those between 0.75 and 0.90 as good, and those greater than 0.90 as excellent [55]. The SEM and MDC95% indicate the measurement error level in the same values as the original measurement (degrees), with the MDC in particular representing the smallest detectable amount of change that cannot be attributed to measurement error [53,56].

The construct validity of the CS proprioception acuity was examined using correlations with relevant questionnaires examining participants’ clinical status (PI-VAS, NDI-GR, TSK-GR, PCS-GR, and FreNAQ-GR) [57]. Correlations were classified as negligible (0.0–0.25), fair (0.25–0.50), moderate to good (0.50–0.75), or good to excellent (>0.75) [53]. The minimum sample size required for the study was calculated taking into account the large number of correlations and adjusting the level of statistical significance based on the Holm–Bonferroni method [58]. Therefore, the minimum sample size for conducting 30 correlations of primary interest (6 proprioception tests × 5 clinical status questionnaires), with an adjusted statistical significance level α = 0.05/30 = 0.00167 to achieve 80% statistical power with a moderate correlation coefficient r = 0.50, was calculated to be n = 56 participants, calculated with a relevant algorithm for correlational studies (https://sample-size.net/correlation-sample-size/, accessed on 31 May 2023) [59].

## 3. Results

### 3.1. Descriptive Statistics

Overall, 59 patients (34 women) with NSCNP who were referred for physical therapy at a private practice participated in this study. The majority of continuous variables (17 out of 23) were normally distributed (*p* > 0.05), apart from pain duration, mean PI-VAS, NDI-GR, PCS-GR, CS-ROM R rotation, and R rotation proprioception; therefore, descriptive data are presented as the mean (SD), maximum, and minimum statistics for all variables. Participants’ demographic characteristics are displayed in Table 1. Each patient had a current pain episode that lasted longer than three months, with a mean (SD) of 25.05 (29.71) months and a median (IQR) of 12 (30) months.

The majority of participants declared a current low (n = 25) or moderate (n = 24) activity level, with few declaring a high (n = 10) activity level. The descriptive statistics of participants’ clinical status from the questionnaires, the CS-ROM, and the CS proprioception acuity measures are displayed in Table 2. Neither the patients’ demographic characteristics (gender included) nor their self-reported activity level significantly influenced any of the variables reported in Table 2; therefore, these did not seem to influence the main variables of interest in this study.

### 3.2. Within-Day Intra-Rater Reliability of CS Proprioception Acuity

The reliability level of the three sequential proprioceptive measurements using the active target reproduction method at 50% of the available CS-ROM in each movement direction, measured on the same day and time by the same examiner, was evaluated. The ICC_2,1_ (95% CI) values were generally good (0.75–0.79) for all movement directions; however, they improved even further (0.76–0.89) if the first of the three consecutive trials was omitted, except for CS flexion proprioception acuity, which remained unchanged (Table 3). The SEM values ranged between 1.97 and 2.94° (1.38 and 3.02° if the first trial was omitted), and the MDC_95%_ ranged between 5.05 and 8.14° (3.83 and 8.38° if the first trial was omitted), generally registering an acceptable trial-to-trial error level (Table 3).

### 3.3. Construct Validity of CS Proprioception Acuity

As is evidenced in Table 4, in general, there were no statistically significant correlations between CS proprioception acuity, measured with the KFORCE Sens^®^, and patient clinical status, measured with an array of questionnaires assessing average and worst pain intensity over the previous week, the NDI-GR, the TSK-GR, the PCS-GR, and the FreNAQ-GR scales. The only fair correlations registered between R side flexion proprioception acuity and the NDI-GR, TSK-GR, PCS-GR, and FreNAQ-GR were not clinically significant when the corrected significance level was considered (α = 0.00167). There were no significant correlations registered between CS-ROM and CS proprioception acuity either.

In addition, the only fair negative correlations registered between CS-ROM and patient clinical status were between mean PI-VAS and two out of six ROM measures (extension and R rotation), between the PCS-GR and R rotation, and between the FreNAQ-GR and R side flexion (Table 5). However, neither of these were statistically significant.

## 4. Discussion

Chronic neck pain is a widely prevalent clinical problem that affects a large proportion of the world population [1,2]. Although there are various methods of assessment and treatment, the connection between clinical symptoms and the corresponding measurements of movement and proprioception remains unclear [14,32]. Several previous studies have shown small-scale yet statistically significantly impaired proprioception acuity in patients with NSCNP compared with healthy individuals of the same age and gender [23,24,33,35,60,61], indicating that proprioceptive deficits are pathological and predispose patients to a higher likelihood of chronicity, possibly due to microtraumas that occur from poor movement control. However, other research investigations have not demonstrated statistically significant differences in proprioception acuity between patients with NSCNP and healthy controls, either in all [31,62] or in the majority [25,32,63] of the measured tests.

Understanding the relationship between proprioceptive measurements and the severity and diversity of patient clinical status can provide valuable insights for the clinical evaluation of patients with NSCNP and aid in the development of more effective treatment approaches [64]. Expanding our knowledge regarding patient evaluation and treatment may require the application of new clinical assessment tools in the population examined [38,39]. Therefore, the aim of this study was to establish the reliability and validity of a new sensor-based electronic goniometer in measuring CS proprioception acuity via the ‘target angle’ reproduction method within the available pain-free CS-ROM in several movement directions.

Two members of the research team were trained in the measurements first and then meticulously collected all the data. These two research team members each examined half of the participants. The normal distribution of the majority of the data was confirmed with the Kolmogorov–Smirnov test. Initial statistical analysis established that there was no influence of gender or other demographic characteristics, or the level of physical activity in relation to CS proprioception angle repositioning measurements, to avoid confounding. The KFORCE Sens^®^ is a portable lightweight sensor that transmits the recorded data to a user-friendly mobile application for immediate storage, making it an easy-to-use system for collecting kinematic data (angles and speeds) in a clinical setting. The tests employed were safe and did not provoke any unnecessary pain or discomfort in participants, as they involved slow-paced, self-regulated movements assessing CS mobility and sensorimotor control (position sense via active angle reproduction). We kept the number of repetitions per test direction low to reduce patient involvement and potential fatigue or pain from prolonged testing, in contrast to previous studies that recommended six repetitions per test direction as optimal [24,65].

The CS proprioception testing methodology followed with this new tool resulted in good measurement accuracy, partly concurring with other similar studies examining reliability in patients with NSCNP with similar but not identical measurement methods employing classic [29,33,60,66,67] or sensor-based technological equipment [24,38,39]. Specifically considering the sensor-based studies [24,38,39], none of them used exactly the same protocol as the one followed in this study, precluding a direct comparison of results. The first study looked at neck proprioception using two different tools: a laser beam device and an inertial measurement unit (IMU) sensor. It included 23 asymptomatic and 20 participants with NSCNP. The average age of the patients was much younger (25.9 years) compared with the age of those included in this study. Four different directions were tested, taking the average of six repetitions per direction, while sitting and standing, and both head-to-neutral and head-to-target tests were included, as well as absolute and constant errors [24]. For those with NSCNP tested while sitting, the AE showed poor to good between-day intra-rater reliability (ICC range: 0.43–0.8 and SEM range: 0.59–2.34°), but with a test–retest timeframe of 14 days [24]. The second study recruited 28 healthy participants to perform both inter- and intra-rater reliability, with the latter having a test–retest timeframe of 7 days. Six movement directions were tested by calculating the AE, taking the average of three repetitions per direction. The AE showed moderate to good between-day intra-rater reliability (ICC range: 0.51–0.77 and SEM range: 0.76–1.59°) [38]. The most recently published study of the three tested 53 patients with NSCNP for intra- and inter-rated reliability. For intra-rater reliability, a test–retest timeframe of 2 days was applied. Again, six movement directions were tested by calculating the AE, taking the average of three repetitions per direction, with the AE showing moderate to good between-day intra-rater reliability (ICC range: 0.56–0.85 and SEM range: 0.39–0.94°) [39]. The advantages of using new technologies in CS proprioception measurements are the use of lightweight, easy-to-apply, but at the same time accurate devices, allowing patients to perform as many trials as possible.

The ICC_2,1_, a two-way random effects absolute agreement single raters/measurements model, was used as a measure of relative reliability in the present study, as single measurements were used to compare measurement accuracy recorded as absolute deviations from a ‘target angle’ set mid-range within the available CS-ROM movement direction individually calculated and set for each of the participants. The SEM and MDC_95%_ were additionally presented as indices of absolute reliability complementing the ICC, as advised [56]. As a primary validation step, though, only sequential measurements obtained at the same time of day and by one examiner were performed in this study. Further testing is required to examine the stability of those measurements over time and in the hands of different examiners who have received similar training [57].

The average CS proprioception values in this study exceeded the previously identified “dysfunctional threshold” of 4.5° [17,21,33] in all movement directions (Table 2 and Table 3). Most studies have reported proprioception AE deviations surpassing this threshold in people with NSCNP [17]. However, there is substantial variability around this cut-off value, with a proportion of patients exhibiting values toward the normative range, too [13]. This variability may suggest that individual factors, such as injury history, current clinical status, physical activity levels, and overall health, may influence proprioceptive ability. Our findings, however, did not confirm this hypothesis for current clinical status or for pain duration. Specifically, the construct validity examination clearly indicated that there were no statistically significant correlations between the CS proprioception acuity measures and patient clinical status. A comprehensive assessment of patient clinical status was performed using questionnaires relevant to the pathology of NSCNP, comprising the PI-VAS, the NDI-GR, the TSK-GR, the PCS-GR, and a relatively new scale measuring impaired body awareness due to chronic pain, the FreNAQ-GR [48,49,50]. 

The expected outcome based on the ‘theory’ would be that the more pain, disability, distorted body self-perception, fear of re-injury, and catastrophizing the patients with CNP had, the greater the disturbance in proprioceptive ability should have been [8]. To explain the lack of correlations, the literature’s inconsistent results should not be ignored, as this relationship may only apply to some patients with CNP. The fact that proprioception acuity was examined within the available and relatively painless CS-ROM, in which patients do not exhibit functional incapacity or disturbed bodily perception and probably neither fear of re-injury nor experience a sense of catastrophizing, may also explain the lack of correlation between the parameters examined. Furthermore, the particular proprioception testing protocols require participants’ full concentration and are thus significantly dependent on fatigue as well as participants’ lack of concentration and distraction during the experimental procedures. Therefore, several factors may have interfered with ‘joint position sense’ testing during the protocol administration. Finally, statistically, the small range of values for both proprioceptive ability and range of motion measurements may have possibly contributed to the non-statistically significant associations between CS proprioception and clinical status.

The limitations of the present study also affect the generalization of the results to some extent. The study’s participants (n = 59, 25 men and 34 women) belonged to a specific age group (25–65 years), with one or several episodes of pain, lasting 3 months or more, of non-traumatic origin. However, the duration of symptoms varied widely among the participants. It may be that patients’ proprioception acuity has to be separately examined in different patient groups [13], possibly according to a pain phenotyping CNP classification scheme [68]. Future studies should also examine the inter-rater and test–retest reliability of the KFORCE Sens^®^ inertial sensor. Furthermore, its responsiveness following physical therapy interventions has to be verified.

Subsequent investigations might have to elucidate the molecular processes that underlie pain and proprioception [14]. Proposals for more effective interventions aimed at the sensorimotor control system or brain plasticity, as well as for new precise movement measures denoting sensorimotor deficit, are also encouraged. Modifications to the tests already employed may have to be made to take into account the effect of the speed of test execution and neck muscles’ fatigue development. A rigorous reevaluation and standardization of the current cervical proprioceptive impairment testing methodologies and related treatments may be necessary.

## 5. Conclusions

The current study confirmed the accuracy of a proprioception measurement protocol in patients with CNP; however, there was no association of proprioception measurements with the severity of clinical status in a sample of patients with CNP referred for physical therapy. Based on the findings of this study, the KFORCE Sens^®^ may be a suitable tool for further research, potentially proving important in the provision of new findings taking into account the speed and quality of movement execution for a more detailed evaluation of CS proprioception.

## Figures and Tables

**Table 1:** Descriptive statistics of participants’ demographic characteristics (n = 59).

	Mean (SD)	Min–Max
Age (y)	46.32 (11.93)	25–65
Height (m)	1.71 (0.09)	1.55–1.90
Body Mass (kg)	74.66 (14.36)	50–112
Body Mass Index (kg/m^2^)	25.22 (3.64)	18.14–35.80
Pain Duration (months)	25.05 (29.71)	3–122

y: years, cm: centimeters, kg: kilograms, m: meters.

**Table 2:** Descriptive statistics of questionnaires, CS-ROM, and proprioception for the six movement directions (n = 59).

	Mean (SD)	Min-Max
Clinical Status Questionnaires		
PI-VAS mean (0–10)	3.98 (1.92)	0–8
PI-VAS max (0–10)	5.63 (2.32)	1–10
NDI-GR (0–100)	26.74 (10.38)	10–52
TSK-GR (17–68)	39.93 (7.06)	26–55
PCS-GR (0–52)	22.91 (9.86)	6–48
FreNAQ-GR (0–36)	15.24 (6.58)	3–30
CS-ROM		
Flexion (°)	45.39 (12.65)	19.60–71.60
Extension (°)	58.03 (13.58)	26.40–86.60
R Side Flexion (°)	39.10 (9.14)	14.8–59.9
L Side Flexion (°)	37.16 (7.86)	17.0–60.2
R Rotation (°)	66.96 (9.80)	30.8–94.2
L Rotation (°)	64.95 (11.21)	18.2–80.6
Proprioception acuity		
Flexion AE (°)	8.20 (5.79)	0.45–31.75
Extension AE (°)	7.63 (5.59)	0.10–30.30
R Side Flexion AE (°)	6.50 (4.21)	0.40–20.30
L Side Flexion AE (°)	6.52 (4.09)	1.10–19.45
R Rotation AE (°)	10.76 (7.10)	0.50–27.85
L Rotation AE (°)	8.41 (6.29)	0.60–31.35

AE: absolute error, CS-ROM: cervical spine range of motion, FreNAQ: Fremantle Neck Awareness Questionnaire, NDI: Neck Disability Index, PCS: Pain Catastrophizing Scale, TSK: Tampa Scale of Kinesiophobia, VAS: visual analog scale, °: degrees, SD: standard deviation, min: minimum, max: maximum, R: right, L: left.

**Table 3:** Descriptive statistics (mean, SDs) and και reliability indices (ICC_2,1_, SEM, MDC_95%_) for all three and the last two sequential proprioception acuity trials with the KFORCE Sens^®^ goniometer (n = 59).

Mean (SD) 1	Mean (SD) 2	Mean (SD) 3	ICC_2,1_ (95% CI)	SEM (°)	MDC_95%_ (°)
Flexion AE					
6.45 (5.48)	8.32 (6.69)	8.10 (5.61)	0.76 (0.65–0.84)	2.81	7.77
_	8.32 (6.69)	8.10 (5.61)	0.76 (0.63–0.85)	3.02	8.38
Extension AE					
5.84 (4.56)	7.89 (5.75)	7.38 (5.80)	0.77 (0.66–0.86)	2.40	6.66
_	7.89 (5.75)	7.38 (5.80)	0.87 (0.79–0.92)	2.06	5.72
R Side Flexion AE					
5.52 (3.63)	6.18 (4.18)	6.82 (4.46)	0.75 (0.65–0.84)	1.97	5.45
_	6.18 (4.18)	6.82 (4.46)	0.89 (0.81–0.93)	1.38	3.83
L Side Flexion AE					
5.71 (3.68)	6.19 (4.00)	6.85 (4.50)	0.79 (0.69–0.86)	1.82	5.05
_	6.19 (4.00)	6.85 (4.50)	0.84 (0.74–0.90)	1.68	4.65
R Rotation AE					
8.28 (5.48)	9.91 (6.53)	11.60 (7.99)	0.77 (0.61–0.86)	2.94	8.14
_	9.91 (6.53)	11.60 (7.99)	0.87 (0.74–0.93)	2.39	6.62
L Rotation AE					
7.83 (5.10)	8.57 (6.17)	8.26 (6.82)	0.79 (0.69–0.86)	2.80	7.77
_	8.57 (6.17)	8.26 (6.82)	0.87 (0.79–0.92)	2.36	6.54

AE: absolute error, SD: standard deviation, °: degrees, R: right, L: left.

**Table 4:** Correlations (Pearson’s) between CS proprioception acuity and measures of patient clinical status (n = 59).

CS Proprioception		PI-VAS Mean	PI-VAS Max	NDI-GR	TSK-GR	PCS-GR	FreNAQ-GR
Flexion	R	−0.09	0.00	−0.07	−0.14	−0.02	−0.03
	*p*	0.51	0.98	0.58	0.30	0.90	0.80
Extension	R	0.02	0.03	0.03	0.14	0.04	0.23
	*p*	0.85	0.80	0.82	0.30	0.77	0.08
R Side Flexion	R	0.16	−0.01	−0.28	−0.31	−0.27	−0.27
	*p*	0.21	0.92	0.03	0.02	0.04	0.04
L Side Flexion	R	0.16	0.15	−0.22	−0.19	−0.12	0.04
	*p*	0.232	0.27	0.09	0.14	0.37	0.77
R Rotation	R	0.133	0.05	−0.06	−0.03	0.10	−0.22
	*p*	0.315	0.69	0.67	0.80	0.47	0.10
L Rotation	R	−0.02	−0.03	−0.17	−0.14	0.02	−0.04
	*p*	0.87	0.81	0.19	0.27	0.85	0.74

CS: cervical spine, FreNAQ: Fremantle Neck Awareness Questionnaire, NDI: Neck Disability Index, PCS: Pain Catastrophizing Scale, TSK: Tampa Scale of Kinesiophobia, VAS: visual analog scale, R: right, L: left.

**Table 5:** Correlations (Pearson’s) between CS-ROM and measures of patient clinical status (n = 59).

CS-ROM		PI-VAS Mean	PI-VAS Max	NDI-GR	TSK-GR	PCS-GR	FreNAQ-GR
Flexion	R	−0.074	−0.003	−0.211	−0.104	−0.221	−0.133
	*p*	0.578	0.981	0.108	0.432	0.093	0.314
Extension	R	−0.265	−0.031	−0.197	−0.063	−0.218	−0.056
	*p*	0.043	0.814	0.136	0.638	0.097	0.674
R Side Flexion	R	−0.176	0.010	0.040	0.167	−0.185	0.254
	*p*	0.182	0.943	0.761	0.205	0.160	0.052
L Side Flexion	R	−0.126	0.039	0.093	0.066	−0.116	0.216
	*p*	0.342	0.767	0.482	0.621	0.381	0.101
R Rotation	R	−0.272	−0.197	−0.189	−0.054	−0.271	0.013
	*p*	0.037	0.135	0.152	0.684	0.038	0.924
L Rotation	R	−0.066	−0.029	−0.035	0.010	−0.082	−0.006
	*p*	0.617	0.827	0.792	0.943	0.539	0.966

CS-ROM: cervical spine range of motion, FreNAQ: Fremantle Neck Awareness Questionnaire, NDI: Neck Disability Index, PCS: Pain Catastrophizing Scale, TSK: Tampa Scale of Kinesiophobia, VAS: visual analog scale, R: right, L: left.

## Data Availability

The data presented in this study are available upon request from the corresponding author. The data are not publicly available due to the applicable data protection law in Greece (Law 4624/2019).
